# Quadrupolar and anisotropy effects on dephasing in two-electron spin qubits in GaAs

**DOI:** 10.1038/ncomms11170

**Published:** 2016-04-15

**Authors:** Tim Botzem, Robert P. G. McNeil, Jan-Michael Mol, Dieter Schuh, Dominique Bougeard, Hendrik Bluhm

**Affiliations:** 1 JARA-Institute for Quantum Information, RWTH Aachen University, D-52074 Aachen, Germany; 2 Institut für Experimentelle und Angewandte Physik, Universität Regensburg, D-93040 Regensburg, Germany

## Abstract

Understanding the decoherence of electron spins in semiconductors due to their interaction with nuclear spins is of fundamental interest as they realize the central spin model and of practical importance for using them as qubits. Interesting effects arise from the quadrupolar interaction of nuclear spins with electric field gradients, which have been shown to suppress diffusive nuclear spin dynamics and might thus enhance electron spin coherence. Here we show experimentally that for gate-defined GaAs quantum dots, quadrupolar broadening of the nuclear Larmor precession reduces electron spin coherence by causing faster decorrelation of transverse nuclear fields. However, this effect disappears for appropriate field directions. Furthermore, we observe an additional modulation of coherence attributed to an anisotropic electronic *g*-tensor. These results complete our understanding of dephasing in gated quantum dots and point to mitigation strategies. They may also help to unravel unexplained behaviour in self-assembled quantum dots and III–V nanowires.

## Abstract (web summary)

Electron spins in semiconductors form a potential basis for quantum information technology however they are strongly affected by interactions with nuclear spins. Here, the authors show how quadrupolar interactions, although suppressing nuclear dynamics, can result in an anisotropic enhancement of electronic decoherence.

## Graphical abstract

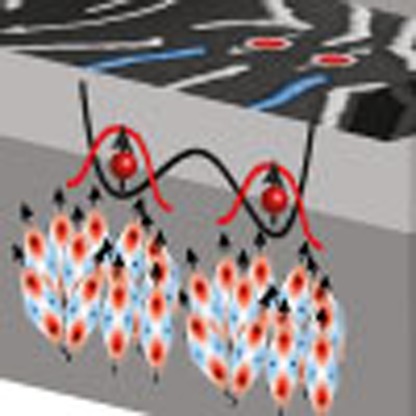

Electron spin qubits in GaAs quantum dots have played a central role in demonstrating the key operations of semiconductor spin qubits^1^,^2^,^3^,^4^. A prominent and often dominant dephasing mechanism in these devices as well as other semiconductor spin qubits^5^,^6^ is the interaction of the electron spin with 10^4^–10^6^ nuclear spins of the host lattice. While the fundamentals of this interaction have been studied quite extensively^7^,^8^,^9^,^10^,^11^, and theory and experiments are in reasonable agreement^12^,^13^, the theory predicts a potential for much longer dephasing times^14^ than observed so far and it remains an open question as to what ultimately limits electron spin coherence. Remarkable progress has also been made in eliminating dephasing from nuclear spins using Si-based systems^15^ that can be isotopically purified, but this route is not open for III–V semiconductor systems, where all isotopes carry nuclear spin. Nevertheless, the latter remain of practical interest because of their lower effective mass, single conduction band valley and potential for optical coupling.

The role of quadrupolar coupling of nuclear spins with electric field gradients (EFGs) from charged impurities or strain has been investigated, both experimentally and theoretically^16^,^17^,^18^,^19^,^20^,^21^, mostly in self-assembled quantum dots, which exhibit large quadrupolar splittings due to strain intrinsic to their epitaxial growth. But its influence on electron spin coherence was unclear and it was first thought to enhance coherence due to quadrupolar suppression of nuclear spin flip-flops.

In contrast to this prediction, we find that Hahn echo coherence of our gate-defined quantum dots deteriorates when the magnetic field is rotated to maximize quadrupolar broadening of nuclear levels. This degradation of coherence is similar to very recent findings in self-assembled quantum dots^22^,^23^, although in our case, quadrupolar splittings arise from local electric fields rather than strain and are orders of magnitude weaker. In addition, we find a complex pattern of collapses and revivals of the echo signal unless the magnetic field is aligned with specific crystal axes, which we explain with an anisotropic *g*-tensor causing a coupling of the nuclear Larmor precession with the electron spin.

## Results

### *S*–*T*_0_ qubit

**Figure 1:**
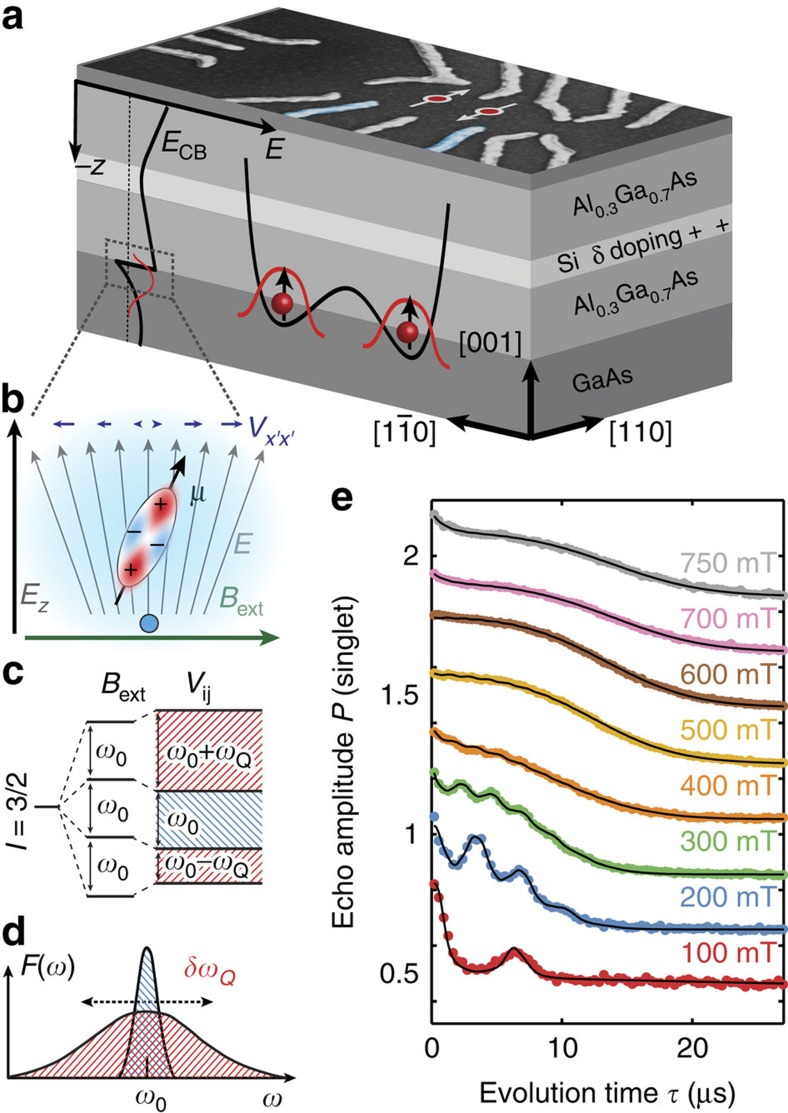
Device layout and quadrupole broadening. (**a**) Gates used for pulsed qubit control are depicted in blue; the energy of the conduction band edge *E*_CB_ is shown on the left. (**b**) Nuclear spins 3/2 with magnetic moment *μ* in the proximity of the quantum dot experience quadrupolar coupling with electric field gradients *V*_*x*′*x*′_ induced by crystal distortion due to the electric field of the triangular quantum well. (**c**) While the centre transition, with splitting *ω*_0_, stays unchanged, the satellite transitions, distorted by the electron's own charge, exhibit a quadrupolar shift by *ω*_*Q*_. (**d**) The resulting frequency distribution *F*(*ω*) consists of two Gaussians with different variances, one showing an excess quadrupolar broadening of *δω*_*Q*_. (**e**) Echo amplitude for magnetic fields along the [110] axis, showing oscillations with the relative Larmor frequencies of the three nuclear spins. A semi-classical model (solid line) is used to fit the data (dots, offset for clarity).

The qubit studied here is a two-electron spin qubit^1^,^24^, using the *m*_*z*_=0 subspace of the spin singlet *S* and spin triplet *T*_0_ of two-electron spins. These electrons are confined in a GaAs double quantum dot formed by electrostatic gating (Fig. 1a) of a two-dimensional electron gas (2DEG). The effects explored in this work apply equally to single electron spins.

A random configuration of the nuclear spins introduces an effective magnetic field of a few mT, the Overhauser field, whose dynamics cause qubit dephasing. Hahn echo measurements that eliminate dephasing from slow fluctuations allow studying these dynamics, as they become the dominant dephasing mechanism.

We follow the experimental procedure from ref. 12 (see also Methods), implementing the required *π*-pulse to invert the state of the qubit halfway through the evolution time 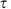, using the exchange interaction between the two spins. Figure 1e shows the spin echo signals as a function of separation time for magnetic fields aligned along the [110] crystal axis. (Note that we experimentally cannot distinguish between the [110] and 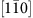 axes, but refer to the direction parallel to the dot connection line as the 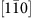 axis throughout the paper for ease of reading.) Similar results to refs 12, 25 are obtained, but with approximately a factor two shorter coherence times (Supplementary Note 1). At fields <500 mT, a second-order coupling with the oscillating, transverse nuclear field (that is, its component perpendicular to the external field) leads to periodic collapses and revivals of the echo amplitude^10^,^11^,^12^,^13^. Revivals occur at times corresponding to the periods of the relative Larmor precession of the three species ^69^Ga, ^71^Ga and ^75^As. The overall envelope decay can be modelled by assuming a phenomenological broadening *δB* of the nuclear Larmor frequencies. Because of this variation of the precession rates, the total transverse hyperfine field of each species decorrelates on the timescale of 1/*δB*. Due to the above-mentioned quadratic contribution of the transverse hyperfine field to the electronic Zeeman splitting, these fluctuations contribute to the dephasing of the electrons.

### Quadrupolar interaction

While such a broadening is expected from dipolar interaction between the nuclei, fitting the current and earlier^12^ data requires a value of *δB*=1.4 and 0.3 mT, respectively, at least a factor three larger than the intrinsic dipolar nuclear linewidth of 0.1 mT obtained from NMR measurements in pure GaAs (ref. 26). More direct measurements of the nuclear dynamics based on correlation of rapid single-shot measurements^27^ are consistent with these values.

NMR experiments on GaAs samples with impurities as well as studies in single self-assembled quantum dots^21^,^28^ revealed a similar excess line broadening, which was found to depend on the field direction and explained by quadrupolar effects^26^,^29^. Strain as well as electric fields from charged impurities or the triangular quantum well, used here to confine electrons (Fig. 1a), distort the valence orbitals and crystal lattice, thus creating EFGs at the nuclear sites (Fig. 1b). These EFGs couple with the quadrupolar momentum of the nuclei with spin *I*=3/2 and (to lowest order) modify the splitting of the *I*_*z*_=±3/2↔±1/2 satellite Larmor transitions by^26^


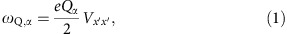


where *Q*_*α*_ is the quadrupolar moment of nuclear species *α*, *e* the elementary charge and *V*_*x*′*x*′_ denotes the component of the EFG tensor in the direction of the external field (Fig. 1b,c). For in-plane fields as considered here, the relevant longitudinal local field gradient induced by an electric field is given by^26^ (Supplementary Note 2)


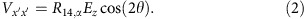


*R*_14,*α*_ is the species-dependent response tensor component relating electric fields to EFGs at the nuclear site due to lattice and orbital distortions, *θ* is the angle between the magnetic field and the [110] axis and *E*_z_ is the electric field component in the *z*-direction. The angular dependence and the fact that only the *z*-component of the electric field contributes, arise from the crystal symmetry of the host material. Hence, the local electric field *E*_*z*_ and its variation across the electronic wavefunction due to the electron's own charge density introduce a broadening of the precession frequencies. The dependence of *ω*_*Q*,*α*_ on *θ*, implies a suppression of the effect for a field along the [100] and [010] axis.

**Figure 2:**
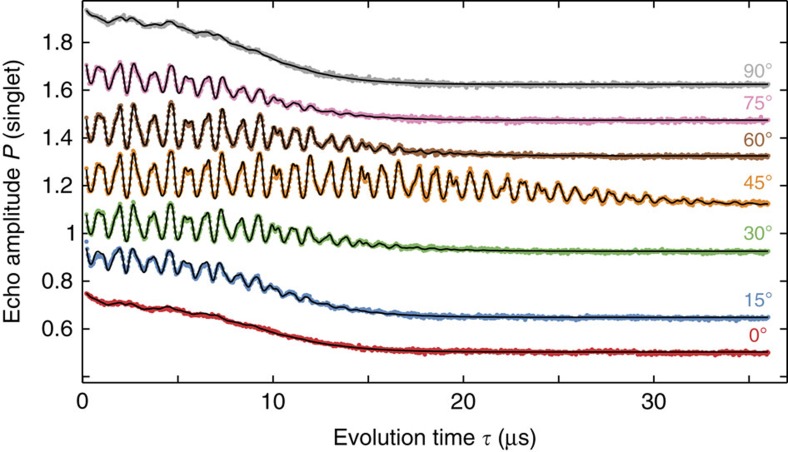
B-field direction dependence. Echo amplitude at 300 mT as a function of separation time for different in-plane magnetic field directions *θ*, with 0° corresponding to the [110] direction. Curves are offset for clarity. At 45°, parallel to the crystallographic [100] axis, the coherence time is enhanced as quadrupolar couplings are suppressed. When rotating the field, a *g*-factor anisotropy leads to oscillations, associated with the three different nuclear Larmor frequencies. A semi-classical model (solid line) is used to fit the data (dots).

The Hahn echo amplitude as a function of separation time is shown in Fig. 2 for different in-plane field directions *θ* between the [110] and the 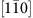 axes (Methods). Indeed, a factor two longer coherence is seen for *θ*=45°, parallel to the [100] (or [010]) direction. Apart from this enhancement, another oscillatory modulation appears, reaching a maximum at the same angle.

### *g*-factor anisotropy

**Figure 3:**
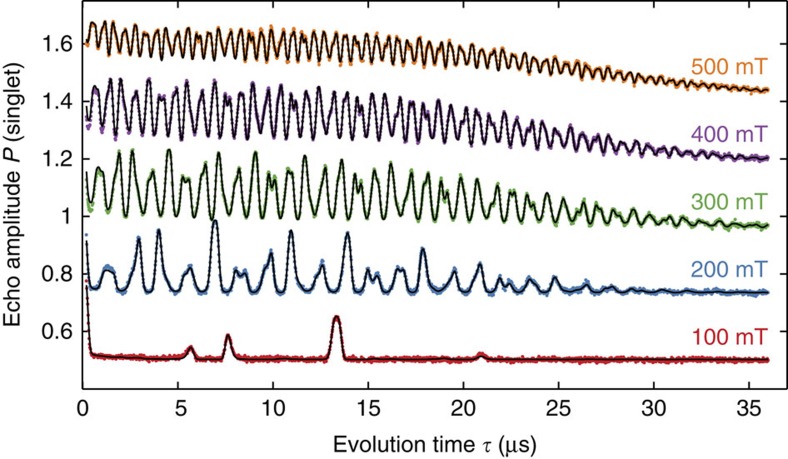
B-field magnitude dependence. Echo amplitude for magnetic field magnitudes along the [100] axis. A *g*-factor anisotropy causing different quantization axes for electron and nuclei spins leads to oscillations with the three nuclear Larmor frequencies. For small magnetic fields, the echo signal is strongly suppressed in the first hundreds of nanoseconds, but revives at later times. A semi-classical model (solid line) is used to fit the data (dots).

**Figure 4:**
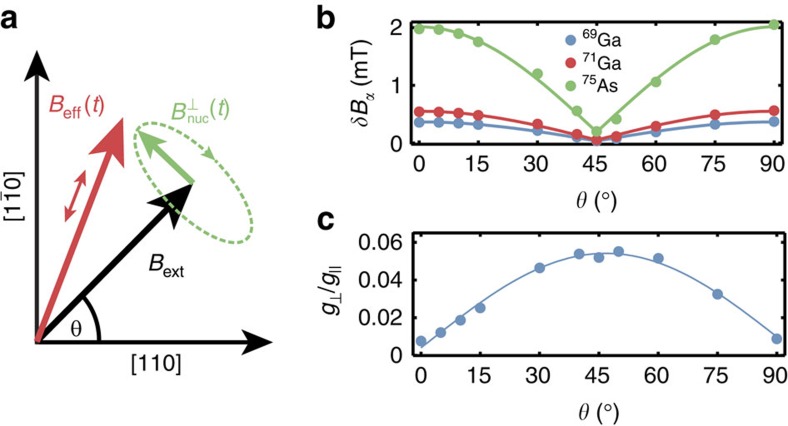
*g*-factor anisotropy and fit parameters. (**a**) Due to an anisotropic *g*-tensor electron and nuclear spins have different quantization axes, *B*_eff_ and *B*_ext_, respectively. This leads to a linear contribution of the transverse Overhauser field to the electronic Zeeman splitting, oscillating with the Larmor frequencies of the nuclear spins. (**b**,**c**) Fit parameters extracted for different in-plane magnetic field directions *θ*. The quadrupolar contribution to nuclear broadening *δB*_*α*_=*ħδω*_*Q*,*α*_/*γ*_*α*_, expressed in terms of an equivalent linewidth for the three isotopes using equation (1), vanishes at 45° along the [100] direction. At the same angle, the sin(2*θ*) dependence of the coupling *g*_⊥_ with the transverse hyperfine field reaches a maximum.

To further investigate the origin of these oscillations, we aligned *B*_ext_ along the [100]-axis and varied its magnitude in Fig. 3. With decreasing *B*_ext_, the frequency of the modulation decreases, until at 100 mT only a very fast decay of the echo amplitude followed by a revival at 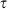
≈13 μs occurs. This envelope modulation can be explained by an electronic *g*-factor anisotropy, arising from an asymmetric confinement of the electron in the 2DEG and spin–orbit coupling^30^,^31^,^32^. The main axes of the *g*-tensor are expected to be the [110] and 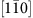 crystal axis, consistent with the absence of a fast echo modulation with *B* along these directions. For other field directions, the quantization axis of the electron differs from the external field around which the nuclear spins precess. A linear coupling with the transverse nuclear magnetic field 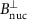 thus appears in the effective magnetic field determining the electronic Zeeman splitting (Fig. 4a; Supplementary Fig. 1; Supplementary Note 3):


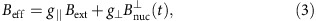


where 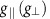 denotes the (off-)diagonal entries of the *g*-tensor. During the free evolution part of the spin echo, the qubit acquires a phase arising from 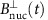. Due to the dynamics of 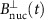 that phase is not eliminated by the echo pulse and hence leads to dephasing. But whenever the evolution time 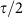 is a multiple of all three Larmor frequencies, the net phase accumulated vanishes and the echo amplitude recovers. Partial recovery occurs if the evolution time only matches a multiple of the Larmor period of two or one species.

### Semi-classical fit model

To obtain a quantitative description of quadrupolar and anisotropy effects, we adapt the semi-classical model of ref. 12, based on computing the total electronic phase accumulated due to the precessing nuclear spins and averaging^13^ over the initial nuclear state. The transverse hyperfine field is modelled as the vector sum of Gaussian distributed contributions arising from the three nuclear species and the spread of quadrupolar shifts. The distribution of nuclear precession frequencies *F*(*ω*) is chosen such that the correlation function of the transverse field is that obtained from an ensemble of independent nuclear spins 3/2 subjected to a Gaussian distribution of quadrupolar shifts (Supplementary Note 4). *F*(*ω*) is taken as the weighted sum of two Gaussians centred on the Larmor frequency, reflecting the contributions from the unperturbed centre transition and the quadrupole broadened satellite transitions as schematically depicted in Fig. 1d. The root mean squared width of the quadrupolar broadened distribution is given by the variation of electric fields via equations (1) and (2).

Using this model, we fit the data (Figs 1, 2, 3) with most free parameters being independent of the magnetic field (Supplementary Note 4).

Most relevant for this work are the quadrupolar broadenings of nuclear transition and the linear coupling with transverse hyperfine fields *g*_⊥_ (both depending on field direction only) shown in Fig. 4b,c. As predicted, the quadrupolar broadening approximately vanishes at *θ*=45° and is maximal at *θ*=0° and *θ*=90°. The maximum magnitude of *δB*_*α*_ is consistent with the electric field variation generated by the electron in the dot (Supplementary Note 4). The off-diagonal *g*-tensor element *g*_⊥_ shows the predicted sin(2*θ*) dependence, and its maximum anisotropy of 5% is comparable with that found in quantum wells^31^.

## Discussion

One of our key results is that quadrupole broadening of nuclear spins can contribute to electronic dephasing by increasing the nuclear linewidth and hence leading to faster decorrelation of the transverse nuclear polarization, which contributes to the electronic Zeeman splitting to second order. While in principle another source of anisotropy with the same angular dependence could explain the observed variation of the coherence time, we are not aware of any other plausible mechanism. Anisotropic diffusion^33^ shows a different angular dependence with the longest coherence times along the [110] direction. Our interpretation is further supported by the good quantitative agreement with the model and NMR measurements^26^,^29^. This result does not contradict the reported suppression of nuclear spin diffusion^20^ by quadrupole effects^19^ as spin diffusion mostly affects electron coherence via the longitudinal polarization, whereas in our case the transverse coupling is dominant. An isotropic *g*-factor in combination with an anisotropic hyperfine interaction would lead to the same echo modulation when rotating *B*_ext_, but the anisotropy of the hyperfine interaction is usually assumed to be negligible as the conduction band wavefunction of GaAs is predominantly s-type.

While in the present sample *g*-factor anisotropy and quadrupolar effects cannot be eliminated simultaneously, symmetric, possibly back-gated quantum wells^31^ should allow the elimination of any *g*-factor anisotropy. The back gate could also be used to tune quadrupolar interaction, as it depends on the electric field, thus allowing further studies.

Given that the strain-induced quadrupole broadening in self-assembled dots was found to be three to four orders of magnitudes larger^19^,^21^,^28^, it likely also has pronounced effects on the coherence^22^ of this type of quantum dot, which is currently less well understood than that of gated dots. In addition to the above-mentioned second-order coupling with the transverse Overhauser fields, a linear coupling of the parallel field components with the effective spin splitting due to the very large and non-uniformly distributed quadrupole splitting in these systems results in a similar, but more complex echo envelope modulation^23^.

Furthermore, the echo modulation due to an anisotropic *g*-factor may also play an important role in III–V nanowire qubits, where strong *g*-factor anisotropies and short coherence times have been measured^34^,^35^.

## Methods

### Qubit system and experimental set-up

The quantum dots used in this work were fabricated on a GaAs/Al_0.69_Ga_0.31_As heterostructure with Si-*δ* doping 50 nm below the surface and a spacer thickness of 40 nm, leaving the 2DEG at 90-nm depth, as shown in Fig. 1a.

Using fast voltage pulses provided by an arbitrary waveform generator (AWG) Tektronix AWG5014C to detune the qubit for manipulation requires thoughtful radio frequency (RF) engineering of the experimental set-up. To avoid any excess pulse distortion, apart from attenuation and skin effect of coaxial cables, we abandon the bias tee and use separate d.c.-coupled static and control gates. Static voltages of order 1 V are applied to the heavily filtered static gates to define and tune the quantum dots. The control gates are used exclusively to apply the mV-scale signals for qubit manipulation. This separation eliminates the need for bias tees and thus provides a nearly flat frequency response of the control gates from d.c. to a few hundred MHz (discussed in Supplementary Note 5 and shown in Supplementary Fig. 2). The control gates are d.c. coupled with the AWG outputs, although heavily attenuated by −33 dBm to reduce thermal noise from room temperature.

### Echo sequence

Following the experimental procedure for Hahn spin echo measurements from ref. 12, we first initialize the qubit system in the spin singlet groundstate *S* by pulsing both electrons into one dot. Rapidly separating the electrons into both dots lets them evolve in different Zeeman fields arising from the external magnetic field *B*_ext_ and the fluctuating local Overhauser field *B*_L(R)_ of the left (right) dot for a time 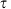. A gradient Δ*B*_*z*_=|*B*_L_−*B*_R_|/2 in the hyperfine field of the two dots leads to coherent rotations between *S* and *T*_0_ and fluctuations in Δ*B*_*z*_ cause dephasing. An exchange splitting between the spin singlet *S* and triplet state *T*_0_ arises from inter-dot tunnel coupling. This exchange allows electric control of the qubit by varying the difference in electrostatic potential between the two dots, on the nanosecond timescale with an AWG. Using this exchange interaction to perform a *π*-pulse by driving rotations between the eigenstates 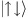 and 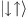, we swap the two electrons halfway through the evolution time 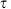. Last, we read out the final qubit state by pulsing the electrons into one dot. Using Pauli spin blockade, we distinguish between the singlet and triplet states by measuring the resistance of a nearby sensing dot via RF reflectometry^36^. Such a pulse cycle with varying evolution times is repeated several million times and the average echo amplitude is recorded. Simultaneous histogramming of individual measurement outcomes is used for normalization^2^ (see Supplementary Figs 2, 3 and 4 and Supplementary Note 6 for details). The fine tuning of the pulses that was necessary in ref. 12 to avoid artefacts from shifts of the wavefunction has been eliminated due to improved RF engineering.

## Additional information

**How to cite this article:** Botzem, T. *et al*. Quadrupolar and anisotropy effects on dephasing in two-electron spin qubits in GaAs. *Nat. Commun.* 7:11170 doi: 10.1038/ncomms11170 (2016).

## Supplementary Material

Supplementary Information Supplementary Figures 1-4, Supplementary Notes 1-6 and Supplementary References
