# Supplementary material for: Quadrupolar and anisotropy effects on dephasing in two-electron spin qubits in GaAs
Source: Nat Commun. 2016 Apr 15;7:11170. doi: 10.1038/ncomms11170 (PMC4835533; doi:10.1038/ncomms11170)
Supplement: Supplementary Information — Supplementary Figures 1-4, Supplementary Notes 1-6 and Supplementary References [file ncomms11170-s1.pdf]

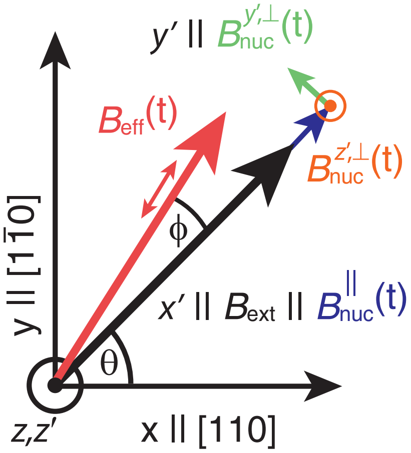


Supplementary Figure 1. B-field directions. This figure visualizes the two different coordinate systems, one is fixed and one rotates with $\mathbf{B}_{\mathrm{ext}}$, and the nomenclature for the relevant field components.

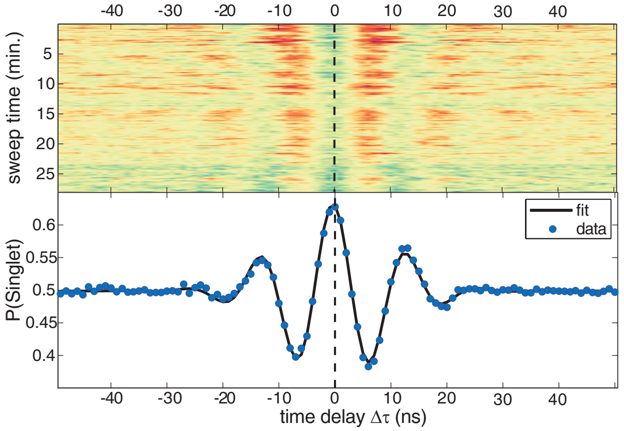


Supplementary Figure 2. Wavefunction shifts. Repetitions of the echo amplitude as a function of a echo pulse time delay $\Delta\tau$ for total evolution time $\tau = 10\,\mu$s at 500 mT. Due to improved high-frequency engineering the position of the maximum echo amplitude does not shift over time, even for stabilized magnetic field gradient $\Delta B_z$, indicating a stable spatial electronic wavefunction over the pulse cycle (compare Ref. 1).

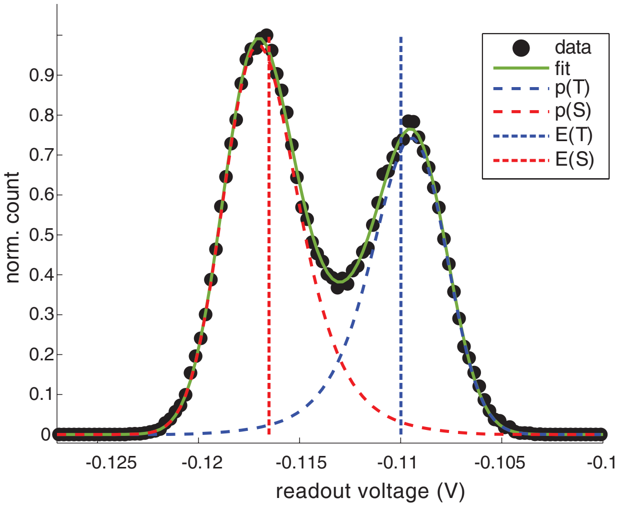


Supplementary Figure 3. Single-shot histogram. Individual measurement outcomes are histogrammed simultaneously with recording the average. By fitting the resulting histogram, we obtain the voltage expectation values $E(S)$ and $E(T)$, representing spin singlet or triplet states. Theses expectation values are used to normalize the averaged measurement data.

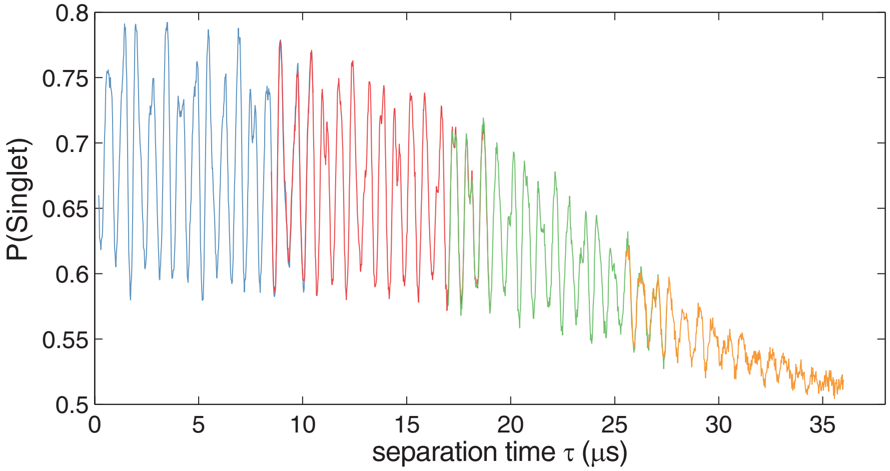


Supplementary Figure 4. Data stitching. Due to limited AWG memory we recorded four individual time traces (different colors) in order to resolve the fast envelope modulation.

## SUPPLEMENTARY NOTE 1. DISCUSSION OF LOWER COHERENCE TIMES

A discussion about the lower coherence times compared to Ref. 1 will be presented in Supplementary Note 5 once the fit model is explained.

## SUPPLEMENTARY NOTE 2. QUADRUPOLAR INTERACTION

In this subsection we derive the broadening of the Larmor frequencies due to dipolar and quadrupolar interaction of a spin 3/2 particle in the presence of a magnetic field and an electric field gradient (EFG) that shifts the normally equidistant Zeeman levels to create three distinct transition frequencies. A distribution of these quadrupolar broadened frequencies leads to dephasing of the qubit. The quadrupole terms are considered to first order in $\omega_{\mathrm{Q},\alpha}/\omega_\alpha \ll 1$, where $\omega_\alpha$ is the Zeeman splitting of species $\alpha$. Our approach is to first compute the two-point time correlator of the transverse Overhauser field from the Zeeman and quadrupole Hamiltonian. We then construct a frequency distribution for the precession of classical Overhauser field contributions resulting in the same correlator.

Following Ref. 6 the Hamiltonian for a spin 3/2 including quadrupolar interaction is

$$\hat{H}_{\mathrm{Q}} = \frac{e^2 qQ}{4I(2I-1)} \left[ 3I_z^2 - I^2 + \eta \frac{\mathbf{I}_+{}^2 + \mathbf{I}_-{}^2}{2} \right], \tag{1}$$

where $Q$ is the quadrupolar moment, $\mathbf{I}$ the spin operator and $\eta = (V_{zz} - V_{y'y'})/V_{x'x'}$ the asymmetry parameter of the electric field gradient tensor $\mathbf{V}$, with spatial derivatives $V_{ij} = \partial^2 V/(\partial r_i \partial r_y)$. Neglecting the non-secular terms because of the large Zeeman splitting, the $m = \pm 3/2 \leftrightarrow m = \pm 1/2$ satellite-transitions frequency shift $\omega_{\mathrm{Q},\alpha}$ only depends on the local EFG $V_{x'x'} = eq$ in the direction of the externally applied magnetic field and we obtain for species $\alpha$[7]

$$\omega_{\mathrm{Q},\alpha} = \frac{eQ_\alpha}{2} V_{x'x'}. \tag{2}$$

In order to relate electric fields $E$ in the sample to EFGs, we use the general tensor relation:

$$V_{ij} = \sum_k R_{ij,k} E_k, \tag{3}$$

where $R_{ijk}$ is the third rank response tensor. The secular component of $\mathbf{V}$ in the $x'$-direction can be written as

$$V_{x'x'} = \hat{\mathbf{n}} \cdot \mathbf{V} \cdot \hat{\mathbf{n}} = -2R_{14,\alpha} \cdot (n_x n_y E_z) \tag{4}$$

$$= 2R_{14,\alpha} \cdot (\cos(\psi)\sin(\psi) E_z)$$

$$= R_{14,\alpha} E_z \sin(2\psi) \tag{5}$$

$$= R_{14,\alpha} E_z \cos(2\theta) \tag{6}$$

with the species dependent response tensor component $R_{14,\alpha}$ and $\hat{\mathbf{n}}$ being a unit vector in the direction of $B_{\text{ext}}$. In the last step the angle $\psi$, measured from the [100]-direction, is transformed to the angle $\theta$, introduced in the last section. We find that the EFG only depends on the electric fields in $z$-direction, which predominantly originates from the triangular quantum well potential of the heterostructure. Nuclear spins in the proximity of the electron quantum dot additionally experience a spatial variation of these EFGs due to the electron's own charge density, which translates into a distribution of quadrupolar frequency shifts.

To obtain the frequency spectrum arising from these shifts we start by deriving the quantum-mechanical correlator of the angular momentum operator. Without loss of generality and consistent with the chosen coordinate system, we consider the $y'$-component of the angular momentum operator of a single spin 3/2

$$\hat{J}_{y'} = \frac{1}{i}\begin{pmatrix} 0 & \sqrt{3} & 0 & 0 \\ -\sqrt{3} & 0 & \sqrt{4} & 0 \\ 0 & -\sqrt{4} & 0 & \sqrt{3} \\ 0 & 0 & -\sqrt{3} & 0 \end{pmatrix}, \tag{7}$$

Using the time evolution operator of the system

$$e^{i\hat{H}t} = \begin{pmatrix} e^{\frac{i\omega_1 t}{\hbar}} & 0 & 0 & 0 \\ 0 & e^{\frac{i\omega_2 t}{\hbar}} & 0 & 0 \\ 0 & 0 & e^{\frac{i\omega_3 t}{\hbar}} & 0 \\ 0 & 0 & 0 & e^{\frac{i\omega_4 t}{\hbar}} \end{pmatrix}, \tag{8}$$

to transform into the Heisenberg picture, one obtains

$$\hat{J}_{y'}(t) = e^{i\hat{H}t}\hat{J}_{y'}e^{-i\hat{H}t} = \tag{9}$$

$$\begin{pmatrix} 0 & \sqrt{3}e^{\frac{i\omega_{12} t}{\hbar}} & 0 & 0 \\ 0 & 0 & \sqrt{4}e^{\frac{i\omega_{23} t}{\hbar}} & 0 \\ 0 & 0 & 0 & \sqrt{3}e^{\frac{i\omega_{34} t}{\hbar}} \\ 0 & 0 & 0 & 0 \end{pmatrix} + \text{h.c.}, \tag{10}$$

where we introduced the transition frequencies $\omega_{ij} = \omega_i - \omega_j$.

Using the infinite temperature density matrix $\rho = \frac{1}{4}\mathbb{1}$ for the spin, we obtain for the two-point correlator

$$\langle \hat{J}_{y'}(t)\hat{J}_{y'}(t=0)\rangle = \text{tr}\left(\hat{J}_{y'}(t)\hat{J}_{y'}(t=0)\rho\right) \tag{11}$$

$$= \frac{3}{8}\cos(\omega_{12}t) + \frac{1}{2}\cos(\omega_{23}t) + \frac{3}{8}\cos(\omega_{34}t). \tag{12}$$

We continue by calculating the correlator for the $y'$-component of the hyperfine magnetic field operator appearing in the nuclear part of the effective Zeeman Hamiltonian $\hat{H}_{\mathrm{nuc}} = g_{\|}\mu_{\mathrm{B}}\hat{\mathbf{B}}_{\mathrm{nuc}}\hat{\mathbf{S}}$:

$$\hat{B}^{y',\perp}_{\mathrm{nuc}}(t) = \sum_i A_i \hat{J}^i_{y'}(t), \tag{13}$$

where $i$ runs over all nuclear spins. Hence

$$\begin{aligned}
&\langle \hat{B}^{y',\perp}_{\mathrm{nuc}}(t)\hat{B}^{y',\perp}_{\mathrm{nuc}}(t=0)\rangle \\
&= \sum_{ij} A_i A_j \langle \hat{J}^i_{y'}(t)\hat{J}^j_{y'}(t=0)\rangle &(14)\\
&= \sum_i A_i^2 \langle \hat{J}^i_{y'}(t)\hat{J}^i_{y'}(t=0)\rangle &(15)\\
&= \sum_i A_i^2 \left(\frac{3}{8}\cos(\omega^i_{12}t) + \frac{1}{2}\cos(\omega^i_{23}t) + \frac{3}{8}\cos(\omega^i_{34}t)\right), &(16)
\end{aligned}$$

where $A_i$ is the hyperfine coupling parameter for spin $i$ and we have used that different spins $i \neq j$ are uncorrelated. The transition frequencies $\omega^i_{23} = \omega_{\alpha(i)} + \Delta\omega_i$ and $\omega^i_{12,34} = \omega_{\alpha(i)} + \Delta\omega_i \pm \omega_{\mathrm{Q},i}$ are composed of the species-dependent Larmor frequency $\omega_{\alpha(i)} = \gamma_{\alpha(i)} B_{\mathrm{ext}}$ with $\gamma_\alpha$ being the gyromagnetic ratio of species $\alpha$, a site dependent frequency shift $\Delta\omega_i = \gamma_{\alpha(i)}\Delta B_i$ arising from an effective field variations $\Delta B_i \approx 0.1$ mT due to interactions between nuclear spins, and the quadrupole shifts $\omega_{\mathrm{Q},i}$. The latter depend on the local electric field via Supplementary Equations (2) and (6) and on the species $\alpha$ via the quadrupole moment $Q_\alpha$. In consideration of the large number of spins, we model these variations by species-dependent continuous frequency distributions

$$F_\alpha(\omega) = \frac{2}{5}\mathcal{N}_{\mathrm{I}}(\omega) + \frac{3}{5}\mathcal{N}_{\mathrm{Q}}(\omega) \tag{17}$$

with $\mathcal{N}_{\mathrm{I}}$ and $\mathcal{N}_{\mathrm{Q}}$ being Gaussian distributions representing the interaction broadened center transition and the quadrupole broadened satellite transitions, respectively. Their prefactors follow from the relative weights in Supplementary Equation (16). $\mathcal{N}_{\mathrm{I}}$ and $\mathcal{N}_{\mathrm{Q}}$ are both assumed to be centered around $\omega_\alpha$. The standard deviation of $\mathcal{N}_{\mathrm{I}}$ is chosen to correspond to an interaction strength $\delta B = 0.1\,\mathrm{mT}$ and that of $\mathcal{N}_{\mathrm{Q}}$ is parametrized in terms of the species independent parameter $\delta\omega_{\mathrm{Q},\alpha}/(Q_\alpha R_{14,\alpha})$. It determines the rms strength of the distribution of local electric field gradients via Supplementary Equation (6), added in quadrature to $\delta B$.

To arrive at a classical description of the dynamics of the nuclear ensemble, we introduce giant effective classical spins $k$ whose contributions to $B_{\text{nuc}}^{\perp}$ are described by uncorrelated Gaussian variables, each representing a narrow range of frequencies of width $\Delta\omega$. We thus express the transverse nuclear field as a sum of complex-valued classical fields $B_{\text{nuc}}^{x',\perp}(t) + iB_{\text{nuc}}^{y',\perp}(t) = \sum_k B_k(t) = \sum_k B_k^x(t) + iB_k^y(t)$. The complex notation was introduced for convenience so that the time-dependence of the $B_k(t)$ terms can be written as

$$B_k(t) = B_k(0)e^{i\omega_k t}. \tag{18}$$

We further write the initial conditions $B_k(0) = \bar{B}_k z_k$ for the $k$th spin in terms of their rms values $\bar{B}_k$ and random variables $z_k = x_k + iy_k$ following a Gaussian probability distribution $p(z, z^*) = \frac{i}{4\pi}\exp\left(-zz^*/2\right)$ with unit variance. The $\bar{B}_k$ reflect both the number of spins with a corresponding level splitting in one of the three transition as well as the strength of that transition. Hence, one can loosely think of each effective spin as representing a group of nuclei with approximately the same transition frequency $\omega_{mn}^i$, with the latter depending on the quadrupolar splitting, the local field arising from dipolar coupling to neighbouring nuclei, and the state of each nucleus determining the available transition.

Equation (18) leads to a correlator

$$\langle \hat{B}_{\text{nuc}}^{y',\perp}(t)\hat{B}_{\text{nuc}}^{y',\perp}(t=0)\rangle = \sum_k \bar{B}_k^2 \cos(\omega_k t). \tag{19}$$

For this correlator to approximate that of Supplementary Equation (16), the $\bar{B}_k$ have to be chosen according to

$$\bar{B}_k^2 = \frac{5}{4}\frac{\mathcal{A}_{\alpha(k)}^2 n_{\alpha(k)}}{N} F_{\alpha(k)}(\omega_k)\Delta\omega. \tag{20}$$

Here $N$ is the number of unit cells overlapping with the quantum dot, $n_{\alpha(k)}$ is the number of nuclei of species $\alpha$ per unit cell and $\mathcal{A}_{\alpha(k)}$ is the hyperfine coupling strength. We neglect a variation of $A_i$ depending on the position of the nuclear spin relative to the electronic wave function as this inhomogeneity of the coupling was found to be negligible in Ref. 1, thus setting $A_i = \mathcal{A}_{\alpha(i)}/N$. We found that discretizing the frequency distribution of each species into seven equidistant intervals $\Delta\omega$ was sufficient to suppress any numerical artifacts.

## SUPPLEMENTARY NOTE 3. $g$-FACTOR ANISOTROPY IN ASYMMETRIC GaAs HETEROSTRUCTURE

In this section we will show that a $g$-factor anisotropy leads to a linear coupling to the transverse Overhauser-field, as illustrated in Supplementary Figure 1, causing additional dephasing (see Supplementary Note 4). In the main axes coordinate system with $x \parallel [110]$, $y \parallel [1\bar{1}0]$ and $z \parallel [001]$, the electronic $g$-tensor is of diagonal form:

$$\underline{\underline{\boldsymbol{g}}} = \begin{pmatrix} g_{xx} & 0 & 0 \\ 0 & g_{yy} & 0 \\ 0 & 0 & g_{zz} \end{pmatrix} . \tag{21}$$

When rotating the external $B$-field by a rotation angle $\theta$ about the [001]-axis we define a new coordinate system, such that $x' \parallel B_{\text{ext}}$, $y' \perp x'$ and $z' = z$. To calculate the $g$-tensor dependence on the rotation angle, we express the $g$-tensor in the rotated basis using the rotation matrix $\mathbf{D}_\theta$

$$\underline{\underline{\boldsymbol{g_\theta}}} = \mathbf{D}_\theta \underline{\underline{\boldsymbol{g}}} \mathbf{D}_\theta^\intercal , \tag{22}$$

For an anisotropic $g$-factor $g_{xx} \neq g_{yy}$, the resulting tensor has both diagonal and off-diagonal components:

$$\begin{aligned} \underline{\underline{\boldsymbol{g_\theta}}} &= \\ &\begin{pmatrix} g_{xx}\cos(\theta)^2 + g_{yy}\sin(\theta)^2 & \frac{1}{2}\sin(2\theta)\left(g_{xx} - g_{yy}\right) & 0 \\ \frac{1}{2}\sin(2\theta)\left(g_{xx} - g_{yy}\right) & g_{xx}\sin(\theta)^2 + g_{yy}\cos(\theta)^2 & 0 \\ 0 & 0 & g_{zz} \end{pmatrix} \\ &= \begin{pmatrix} g_\parallel & g_\perp & 0 \\ g_\perp & g_{y'y'} & 0 \\ 0 & 0 & g_{zz} \end{pmatrix} . \end{aligned} \tag{23}$$

In the last step we have defined the components parallel and perpendicular to $B_{\text{ext}}$ as

$$g_\parallel = g_{x'x'} = g_{xx}\cos(\theta)^2 + g_{yy}\sin(\theta)^2 \approx g_{xx}, \tag{24}$$

$$g_\perp = g_{x'y'} = g_{y'x'} = \frac{1}{2}\sin(2\theta)\left(g_{xx} - g_{yy}\right), \tag{25}$$

and find that the perpendicular components show a $\sin(2\theta)$ dependence.

With this $g$-factor anisotropy the Hamiltonian of the system now states

$$\hat{\mathbf{H}} = \mu_{\mathrm{B}} \underline{\underline{\boldsymbol{g_\theta}}} \mathbf{B}_{\mathrm{ext}} \cdot \hat{\mathbf{S}} + \mu_{\mathrm{B}} g_{\|} \mathbf{B}_{\mathrm{nuc}} \cdot \hat{\mathbf{S}} \tag{26}$$

$$= \mu_{\mathrm{B}} \left( \underline{\underline{\boldsymbol{g_\theta}}} \mathbf{B}_{\mathrm{ext}} \mathbf{O}^{\mathsf{T}} \right) \cdot \mathbf{O}\hat{\mathbf{S}} + \mu_{\mathrm{B}} g_{\|} \mathbf{B}_{\mathrm{nuc}} \cdot \mathbf{O}^{\mathsf{T}} \mathbf{O} \hat{\mathbf{S}}. \tag{27}$$

Note that the choice of $g_{\|}$ to convert the hyperfine coupling to an effective magnetic field is a matter of convention. In the second step we have defined

$$\mathbf{O} = \begin{pmatrix} \cos(\phi) & -\sin(\phi) & 0 \\ \sin(\phi) & \cos(\phi) & 0 \\ 0 & 0 & 1 \end{pmatrix}, \tag{28}$$

such that the vector $\underline{\underline{\boldsymbol{g_\theta}}} \mathbf{B}_{\mathrm{ext}} \mathbf{O}^{\mathsf{T}}$ only has one nonzero component along the effective quantization direction of the electron. $\phi$ is the angle between $\mathbf{B}_{\mathrm{ext}}$ and the electron quantization axis $\underline{\underline{\boldsymbol{g_\theta}}} \mathbf{B}_{\mathrm{ext}}$ and $\hat{\mathbf{S}}$ denotes the spin operator. With $\mathbf{O}\hat{\mathbf{S}} = \tilde{\mathbf{S}} = (\tilde{S}_x, \tilde{S}_y, \tilde{S}_z)$ and $\mathbf{B}_{\mathrm{nuc}} = (B^{\|}_{\mathrm{nuc}}, B^{y',\perp}_{\mathrm{nuc}}, B^{z',\perp}_{\mathrm{nuc}})$ we write, ignoring non-secular terms

$$\begin{aligned} \hat{H} = {} & \mu_{\mathrm{B}} \sqrt{g_{\|}^2 + g_{\perp}^2} B_{\mathrm{ext}} \tilde{S}_x \\ & + \mu_{\mathrm{B}} g_{\|} \left( B^{\|}_{\mathrm{nuc}} \cos(\phi) + B^{y',\perp}_{\mathrm{nuc}} \sin(\phi) \right) \tilde{S}_x. \end{aligned} \tag{29}$$

We further include the second-order coupling to the hyperfine field component perpendicular to the quantization axis $\underline{\underline{\boldsymbol{g_\theta}}} \mathbf{B}_{\mathrm{ext}}$, derived in Refs. 1 and 5, and obtain for the total Hamiltonian

$$\begin{aligned} \hat{H} = {} & \mu_{\mathrm{B}} \sqrt{g_{\|}^2 + g_{\perp}^2} B_{\mathrm{ext}} \tilde{S}_x \\ & + \mu_{\mathrm{B}} g_{\|} \left( B^{\|}_{\mathrm{nuc}} \cos(\phi) - B^{y',\perp}_{\mathrm{nuc}} \sin(\phi) \right) \tilde{S}_x \\ & + \mu_{\mathrm{B}} g_{\|} \frac{\left( B^{y',\perp}_{\mathrm{nuc}} \cos(\phi) + B^{z',\perp}_{\mathrm{nuc}} - B^{\|}_{\mathrm{nuc}} \sin(\phi) \right)^2}{2B_{\mathrm{ext}}} \tilde{S}_x \\ \approx {} & \mu_{\mathrm{B}} g_{\|} \left( B_{\mathrm{ext}} + B^{\|}_{\mathrm{nuc}} - \frac{g_\perp}{g_{\|}} B^{y',\perp}_{\mathrm{nuc}}(t) + \frac{B^{\perp}_{\mathrm{nuc}}(t)^2}{2B_{\mathrm{ext}}} \right) \tilde{S}_x. \end{aligned} \tag{30}$$

In the last step we assumed the anisotropy to be small such that $\sin\phi \approx \tan\phi = g_\perp / g_{\|}$ and $\cos(\phi) \approx 1$. Furthermore, we have defined $(B^{\perp}_{\mathrm{nuc}})^2 = (B^{y',\perp}_{\mathrm{nuc}})^2 + (B^{z',\perp}_{\mathrm{nuc}})^2$. We thus find that an anisotropy in the electric $g$-factor leads to linear coupling to the transverse hyperfine-field. However, note that an anisotropic hyperfine coupling tensor in the second term of Supplementary Equation (26) would lead to the same Hamiltonian and could not

be distinguished from a $g$-factor anisotropy based on our data. We interpret our results in terms of the latter as such an anisotropy is known to exist, whereas we are not aware of any known corrections to the isotropic contact hyperfine interaction for the s-type GaAs conduction band.

## SUPPLEMENTARY NOTE 4. SEMI-CLASSICAL FIT MODEL

### A. Derivation

In this subsection we derive the fitmodel used to fit all data sets in the main text. The model is a generalization of the semi-classical approach used in Refs. 1 and 5 as it accounts for a linear coupling to the transverse field component via a $g$-factor anisotropy from Supplementary Note 3 and quadrupolar broadening of the individual Larmor frequencies from Supplementary Note 2.

Following Supplementary Equation (30), the effective Zeeman splitting is given by

$$E_{\mathrm{Z}}(t) = g_{\parallel}\mu_{\mathrm{B}}\left(B_{\mathrm{ext}} + \frac{B_{\mathrm{nuc}}^{\perp}(t)^2}{2B_{\mathrm{ext}}} + \frac{g_{\perp}}{g_{\parallel}}B_{\mathrm{nuc}}^{\perp,y}(t)\right), \tag{31}$$

including both quadratic and linear coupling to the perpendicular nuclear magnetic fields arising from the three species $^{69}$Ga, $^{71}$Ga and $^{75}$As.

Introducing the giant spin model from Supplementary Equation (18), the relative phase pickup $\Phi$ for a separation time $\tau$, given initial conditions $B_k(0)$ and for one quantum dot for the Hahn echo ($c(t) = 1$ $(-1)$ for $t < \tau/2$ $(t > \tau/2)$) is

$$\begin{aligned}
&\Phi(\tau) = \\
&\frac{\mu_{\mathrm{B}}}{\hbar}\int_0^{\tau} c(t)\left(\frac{g_{\parallel}}{2B_{\mathrm{ext}}}\sum_{k,l} B_k(t)B_l^*(t) + g_{\perp}\sum_k \Re\left(B_k(t)\right)\right)dt \\
&= \frac{g_{\parallel}\mu_{\mathrm{B}}}{2\hbar B_{\mathrm{ext}}}\sum_{k,l}\bar{B}_k\bar{B}_l z_k z_l^* \int_0^{\tau} c(t)e^{i\omega_{kl}t}dt + \\
&\quad g_{\perp}\frac{\mu_{\mathrm{B}}}{\hbar}\sum_k \bar{B}_{\mathrm{k}}x_{\mathrm{k}}\int_0^{\tau} c(t)\Re\left(e^{i\omega_k t}\right)dt \qquad (32) \\
&= \sum_{k,l} T_{k,l}\frac{z_k z_l^*}{2} + \sum_k b_k x_k. \qquad (33)
\end{aligned}$$

In the second step we replaced the initial conditions by the random numbers $z_k$ and the rms-values from Supplementary Equation (20). Furthermore $\omega_{kl} = \omega_k - \omega_l$ and $b_{\mathrm{k}} = g_\perp \frac{\mu_{\mathrm{B}}}{\hbar} \bar{B}_k$. The definition of $T_{kl}$ follows from the last equality and is consistent with Ref. 1.

To perform the ensemble average $\langle e^{-i\Phi} \rangle$ we have to integrate over the distribution of initial nuclear fields. This integral is solved for one single electron dot by applying the $T$-matrix approach, similar to Ref. 1, extended by a linear coupling term $b_k x_k$.

$$\begin{aligned}\langle e^{-i\Phi}\rangle = &\int \left(\prod_j dz_j dz_j^* p(z_j, z_j^*)\right) \times \\ &\exp\left(-i\sum_{k,l} T_{kl}\frac{z_k^* z_l}{2} + \sum_k b_k x_k\right) \qquad (34)\\ &= \prod_j \int dz_j dz_j^* p(z_j, z_j^*) \cdot \exp\left(-i\lambda_j \frac{|z_j|^2}{2} + \tilde{b}_j x_j\right) \\ &= \prod_j \frac{1}{1+i\lambda_j} \exp\left(\frac{2\tilde{b}_j^2}{1+i\lambda_j}\right), \qquad (35)\end{aligned}$$

where $\lambda_j$ are the eigenvalues from $T_{k,l} = UD_{k,l}U^\dagger$ for group $j$, with $D$ being the diagonal matrix of eigenvalues $\lambda_j$. For $\tilde{b} = Ub$ the same basis transformation is performed.

As the nuclei sampled by the electron spins in the left and right dot can be assumed to be statistically independent, the total decoherence function for dephasing of the two electron spins in a double dot can be written as the product of two identical dots $|\langle e^{-i\Phi}\rangle|^2$. While for an ideal Hahn echo measurement the echo response is given by Supplementary Equation (35), we also included a visibility factor $\mathcal{N}$ and an overall offset $o$ in our fit model to account for imperfect $\pi$-pulses in the refocusing part of the Hahn echo sequence, loss of measurement contrast at higher magnetic fields and possible leakage.

The final fit function of the Hahn echo amplitude states

$$\begin{aligned}P_{\mathrm{S}}(\tau) = &\mathcal{N}\prod_j \left(\frac{1}{1+i\lambda_j}\exp\left(\frac{2\tilde{b}_j^2}{1+i\lambda_j}\right)\right)^2 e^{\left(-\frac{\tau}{T_{\mathrm{SD}}}\right)^{-4}} \\ &+ o + Ae^{-\frac{\tau}{1.5\mu\mathrm{s}}}. \qquad (36)\end{aligned}$$

Here we also introduced a multiplicative term to account for dephasing due to spectral diffusion, which according to theory[8,9] shows a $e^{-(\tau/T_{\mathrm{SD}})^{-4}}$ dependence (see Sec. C). Furthermore, we add an additional exponential decay with a fixed time constant of $1.5\,\mu$s (see Sec. D). Using this model we perform a global fit of all measurement data (Fig. 1-3 of the main

text) with the following free parameters: the number of unit cells $N$, the spectral diffusion time constant $\tau_{\mathrm{SD}}$ (see Sec. C), the species independent parameter $\delta\omega_{\mathrm{Q},\alpha}/(Q_\alpha R_{14,\alpha})$ related to the distribution of EFGs (see Supplementary Note 2 and Sec. B), the linear coupling to transverse hyperfine fields $g_\perp$ and the scaling and offset parameter (allowed to vary for each data set). The value for $N$ was kept unchanged for all data sets and the best fit yields a value of $2.4 \times 10^6$ nuclear spins, with two electrons per unit cell for GaAs.

### B. Variance of electric field

Modeling the electron as a two-dimensional $25 \times 25$ nm charge density we estimate a maximal change of the EFG of $\delta(\partial E/\partial z) = R_{14,\alpha} e/(\epsilon_0 \epsilon_r 25\ \mathrm{nm}^2)$ across the wave function according to the Poisson equation and Supplementary Equation (6). Expressing this EFG as equivalent Larmor line width using Supplementary Equation (2) and using literature values, we obtain broadenings reaching from 0.34 to 1.86 mT for the different species, in good agreement with the fitted values. Even though we can reproduce the line width quite well, other effects, such as strain, electrical field from charged impurities and the lateral confining gates may also contribute to quadrupolar interaction.

### C. Spectral Diffusion Time Constant

The spectral diffusion term introduced in Supplementary Equation (36) accounts for additional dephasing from fluctuations of $B_{\mathrm{nuc}}^{\parallel}$ due to spin diffusion. The multiplicative form can be justified with the assumption that $B_{\mathrm{nuc}}^{\parallel}$ and $B_{\mathrm{nuc}}^{i,\perp}$ are uncorrelated, with $i$ being either $y'$ or $z'$. The value of $\tau_{\mathrm{SD}}$ is difficult to extract independently without going to very large magnetic fields. We obtain good fits by fixing it at $\tau_{\mathrm{SD}} = 30\,\mu$s for all data sets, consistent with earlier measurements[1] and theory. As the resulting decay sets in at larger times than that seen in the experimental traces, this value corresponds to a very small effect on the fitted curves and indicates that spectral diffusion has a very small contribution compared with dephasing from transverse fields. However, the quality of the fits is not very sensitive to $\tau_{\mathrm{SD}}$ so that the above value is not very reliable.

### D. Deviation of First Data Points

Similar to Ref. 1 we find that the first data points deviate from our fit model and our best fits show an exponential time dependency with a time constant of $1.5\,\mu$s with varying amplitude. This additional decay is most visible for high values of the external magnetic field and also more pronounced with a high Overhauser field $\Delta B_z$, i.e. the echo time trace at 750 mT in Fig. 1c of the main text. While the origin of this behaviour is currently not understood, it was phenomenologically added to the fit model to ensure that deviations at short times do not affect the quality of the overall fit.

### E. Discussion of Lower Coherence Times

The size of the quantum dot determines the overlap of the electron wavefunction with the nuclear spins in the vicinity and hence has a strong influence on coherence times. Fitting all our data, we find that the number of overlapping nuclei is a factor of two smaller compared with Ref. 1, leading to shorter coherence times, that are more comparable with Ref. 10. Note that a smaller electronic wavefunction directly translates into an increased quadrupolar broadening $\delta B_\alpha$ via Supplementary Equation (2) and (6), further contributing to lower coherence time. The smaller wavefunction may be caused by the thinner spacer layer of the heterostructure and possibly more disorder in the heterostructure used in this work, compared with the one used in Ref. 1.

### F. Loss of Visibility

The $\pi$-pulse used to invert the qubit state is realized by detuning the electrons for a typical time of $5-7$ ns to a finite exchange splitting $J$, which was adjusted to show maximal contrast before each measurement run. Ideally, the $\pi$-pulse should rotate about a Bloch sphere axis perpendicular to that of $\Delta B_z$, which is only achieved for $J \gg \Delta B_z$. As we cannot set the gradient $\Delta B_z$ to zero, the two rotation axes are in practice not perfectly orthogonal. We typically used an exchange value of $J \approx 160$ MHz and limited $\Delta B_z <$ 60 MHz, limiting the typical contrast of the Hahn echo to a value of around 0.3. The gradient $\Delta B_z$ is induced unintentionally and for unknown reasons by the pulses applied to the qubit. For measurements performed at magnetic fields above 500 mT the induced

polarization increased, for an unknown reason so far, further reducing the visibility due to faster relaxation during the readout of the qubit state[11].

## SUPPLEMENTARY NOTE 5. CHECKS FOR SLOW DRIFTS IN THE CONTROL ELECTRONICS

To check for slow drifts in the control pulses, which would lead to spatial shifts of the electronic wavefunction (compare Ref. 1), we shift the $\pi$-pulse by a time $\Delta\tau$ and track the echo amplitude as a function of $\Delta\tau$ of the $\pi$-pulse for the whole measurement time. For $\Delta\tau = 0$ the evolution time before and after the $\pi$-pulse is exactly the same and should yield the best refocusing. A drift of the maximum of the echo amplitude would indicate that the electrons sample different nuclear spins before and after the $\pi$-pulse, caused for example by a change in gate voltages. In Supplementary Figure 1 one can clearly see that the position of the echo amplitude is stable over the whole measurement time, even for intentionally stabilized $\Delta B_z$ which showed to increase the shift in position[1], reflecting a stable control voltage over the duration of the separation time. Nonetheless we tested the effect of the pulse optimization procedure from Ref. 1 by adding a slope to the detuning once the electrons are separated but are not able to obtain longer coherence times. This test confirms that the pulses already have the optimal shape without further compensation.

## SUPPLEMENTARY NOTE 6. DATA NORMALIZATION AND STITCHING

In order to link measured RF sensor[2] voltages to the actual qubit states $|S\rangle$ and $|T_0\rangle$ we simultaneously record histograms of the single shot readout voltage, averaged over the measurement window after each pulse. As seen in Supplementary Figure 3 these histograms typically show two Gaussian distributions, corresponding to $|S\rangle$ and $|T_0\rangle$, which are smeared out due to $T_1$ relaxation from $|T_0\rangle$ to $|S\rangle$ and excitation from $|S\rangle$ to $|T_0\rangle$. Using a fit model similar to Ref. 3 and a measured relaxation time of $\approx 16\,\mu$s for the metastable (1,1) charge configuration, we are able to obtain the distributions for singlet and triplet states. (Note that this relaxation occurs at the measurement point and is different from the relaxation rate of the qubit at its operating point with separated electrons.) We then use the mean voltages $E(S)$ and $E(T)$, corresponding to S and T values, respectively, to normalize our

measurement data. While this procedure should largely eliminate readout-related visibility losses, $\pi$-pulse errors still contribute to the visibility being less than 0.5. As the relaxation time shows a strong dependence on the magnetic field gradient $\Delta B_z$, we restrict our measurements to gradients below 60 MHz by using Dynamic Nuclear Polarization (DNP)[4]. Simultaneously recorded values for $\Delta B_z$ allow for post-selection in case DNP failed (only used for measurements performed at high external magnetic fields along the [110]-axis).

To resolve the fast oscillations of the echo amplitude when rotating the external magnetic field, it is necessary to increase the resolution of the evolution time $\tau$ to 20 ns, exceeding AWG memory for $\tau = 36\,\mu$s if all pulses were uploaded simultaneously. We therefore consecutively record four different time intervals, each overlapping by $2\,\mu$s, and stitch them back together post measurement. Recording individual single shot histograms and normalizing data before stitching allows the removal of slow $1/f$-noise and individual time traces fit well, as depicted in Supplementary Figure 4. Within each interval, a low-noise, high data quality is obtained by recording different evolution times consecutively and then averaging over many repetitions to elude slow drifts in the sensor or gate voltage configuration. For a typical dataset the evolution time was rapidly swept 101 times for a total measurement time of $101 \times 36\,\mu\text{s} \approx 3.6\,\text{ms}$ and then averaged over $> 250$ repetitions.

## SUPPLEMENTARY REFERENCES


1 Bluhm, H. *et al.* Dephasing time of GaAs electron-spin qubits coupled to a nuclear bath exceeding 200$\mu$s. *Nature Physics* **7**, 109–113 (2011).

2 Reilly, D. J., Marcus, C. M., Hanson, M. P. & Gossard, A. C. Fast single-charge sensing with a rf quantum point contact. *Applied Physics Letters* **91**, 162101 (2007).

3 Barthel, C., Reilly, D. J., Marcus, C. M., Hanson, M. P. & Gossard, A. C. Rapid Single-Shot Measurement of a Singlet-Triplet Qubit. *Physical Review Letters* **103**, 160503 (2009).

4 Bluhm, H., Foletti, S., Mahalu, D., Umansky, V. & Yacoby, A. Enhancing the Coherence of a Spin Qubit by Operating it as a Feedback Loop That Controls its Nuclear Spin Bath. *Physical Review Letters* **105**, 216803 (2010).

5 Neder, I. *et al.* Semiclassical model for the dephasing of a two-electron spin qubit coupled to a coherently evolving nuclear spin bath. *Physical Review B* **84**, 035441 (2011).

[6] Suits, B. H. Nuclear Quadrupole Resonance Spectroscopy. In *Handbook of Applied Solid State Spectroscopy* (Springer US).

[7] Sundfors, R. K. Exchange and Quadrupole Broadening of Nuclear Acoustic Resonance Line Shapes in the III-V Semiconductors. *Physical Review* **185**, 458–472 (1969).

[8] Witzel, W. M. & Das Sarma, S. Quantum theory for electron spin decoherence induced by nuclear spin dynamics in semiconductor quantum computer architectures: Spectral diffusion of localized electron spins in the nuclear solid-state environment. *Physical Review B* **74**, 035322 (2006).

[9] Yao, W., Liu, R.-B. & Sham, L. J. Theory of electron spin decoherence by interacting nuclear spins in a quantum dot. *Physical Review B* **74**, 195301 (2006).

[10] Medford, J. *et al.* Scaling of Dynamical Decoupling for Spin Qubits. *Physical Review Letters* **108**, 086802 (2012).

[11] Barthel, C. *et al.* Relaxation and readout visibility of a singlet-triplet qubit in an Overhauser field gradient. *Physical Review B* **85**, 035306 (2012).
